# Emerging Illicit Drug “2C”: A Case Report on Its Hallucinogenic and Stimulant Properties

**DOI:** 10.7759/cureus.69407

**Published:** 2024-09-14

**Authors:** Marina Nasr, Brian Assadi, Brianna C Weissman, Olyvia Gleason, Jacqueline Seikunas, Manoj Puthiyathu

**Affiliations:** 1 Psychiatry, St. George's University School of Medicine, True Blue, GRD; 2 Medicine, St. George's University School of Medicine, True Blue, GRD; 3 Psychiatry, University of New England, Biddeford, USA; 4 Psychiatry, Bergen New Bridge Medical Center, Paramus, USA

**Keywords:** 2c, bipolar disorder and drug use, emerging drugs, hallucinogenic, party drug

## Abstract

“2C,” formally known as 4-bromo-2,5-dimethoxyphenethylamine, is an illicit drug that combines elements of ketamine, MDMA (ecstasy), methamphetamine, cocaine, and opioids. This report highlights the emergence of 2C compounds, a new class of illicit drugs recognized for their distinctive blend of hallucinogenic and stimulant properties. We present the case of a 22-year-old female who was admitted to the psychiatric emergency department with a history of bipolar I disorder and recent use of various illicit substances, including the drug known as 2C. The patient exhibited symptoms such as visual hallucinations, euphoria, and an increased heart rate. Laboratory tests and toxicology screens were performed to confirm the presence of the components associated with the 2C compound. Her management involved admission to an acute inpatient psychiatric unit for medication stabilization. This case underscores the critical need for healthcare providers to recognize the signs and symptoms of 2C compound intoxication and to provide timely, appropriate intervention. With the rise in recreational use of such substances, further research and public health initiatives are essential to address the associated risks.

## Introduction

In the past decade, the rapidly evolving party scenes, especially within electronic dance music events in the United States, have been matched by a notable shift in the psychoactive substances used. The drug market has expanded quickly, introducing a range of new and unfamiliar mixtures. Historically, cocaine and ketamine have been popular choices [1]. However, the emergence of designer drugs has led to the synthesis of numerous new compounds, each with unique psychoactive profiles [2]. Among these, 4-bromo-2,5-dimethoxyphenethylamine, commonly known as 2C/2C-B or colloquially as “pink cocaine,” has gained increasing attention [3,4]. This designer drug exhibits effects similar to those of ketamine, MDMA (ecstasy), methamphetamine, cocaine, and occasionally opioids, resulting in a diverse range of side effects, including agitation, aggression, anxiety, confusion, hallucinations, hypertension, mydriasis, and tachycardia [5-8].

The drug’s effects on neurotransmitter systems vary, with some formulations showing MDMA-like effects due to the inhibition of noradrenaline and serotonin reuptake and partial agonism at 5-HT-2A and 5-HT-2B receptors. More potent variants, such as the NBOMe, exhibit highly selective agonistic effects on 5-HT-2A/5-HT-1A receptors and an affinity for alpha-1 adrenergic receptors, contributing to its strong hallucinogenic effects and extensive side effect profile [9-11].

Given the drug’s rare prevalence - with only 34 cases reported in 2023 - and its impact on mood and emotions, inducing both hallucinogenic and sympathetic alterations, we explored its implications in a patient with a history of mood and psychiatric disorders [3]. Due to the varying compositions of this designer drug, standard drug screens may not detect it accurately, potentially delaying diagnosis. Considering the drug’s polysubstance nature and its popularity among younger users, this report aims to highlight the temporal and sequelae effects on vulnerable populations.

## Case presentation

We relay the case of a 22-year-old African American female with a history of bipolar I disorder, most recently presenting with severe depressive symptoms, anxiety, suicidal ideations without prior attempts, and self-injurious behavior involving superficial lacerations. Her medication regimen, prescribed by her outpatient psychiatrist 3.5 weeks before presentation, included aripiprazole 5 mg daily, fluoxetine 20 mg daily, hydroxyzine HCL 25 mg daily as needed for anxiety, lamotrigine 25 mg daily, and propranolol 10 mg daily as needed for panic. However, the patient reported nonadherence to her medication regimen and failure to follow up with her psychiatrist for medication stabilization.

The patient arrived at the emergency department with her mother after an episode of agitation and aggression following the use of 2C the previous night, seeking further psychiatric help. At presentation, her primary complaints included worsening mood symptoms, recent manic behavior characterized by impulsivity, irritability, and increased risk-taking. She appeared anxious and guarded with a constricted effect but was otherwise calm, cooperative, and not in acute distress. She denied both suicidal and homicidal ideations, intents, or plans, and also denied experiencing auditory or visual hallucinations. The patient reported frequent recreational substance use, including cocaine two to three times per week for the past month, and recent use of 2C, a substance combining ketamine, cocaine, and MDMA. Urine toxicology tested positive for cocaine, and the patient admitted to using 2C. Consequently, she was admitted to the acute inpatient psychiatric unit for bipolar I disorder with a severe depressive episode and cocaine use disorder.

In the inpatient psychiatric unit, the initial treatment plan included quetiapine 50 mg in the morning and 100 mg at night to address mood lability, agitation, and irritability. As-needed medications included haloperidol 5 mg every six hours for agitation, lorazepam 2 mg every six hours for anxiety, and diphenhydramine 50 mg every six hours for extrapyramidal symptoms. On the second day, the rapid response team was called due to a hypotensive episode with symptoms of weakness and dizziness; the patient’s blood pressure was 81/49 with a heart rate of 96 beats per minute. She was evaluated and managed on the unit, with her treatment plan adjusted. Quetiapine was discontinued due to symptomatic hypotension, and lamotrigine was initiated, given its positive response in the past. Olanzapine 2.5 mg was started at night for further management of mood symptoms. Over the remainder of her six-day hospitalization, the patient adhered to her medication regimen, showing symptomatic improvement. Lamotrigine and olanzapine were up-titrated to 50 mg twice a day and 5 mg at night, respectively. With adherence, the patient’s mood improved significantly, and she experienced notable reductions in irritability and agitation. Her final diagnosis at discharge was bipolar I disorder, a most recent episode of depression with mixed features, severe.

## Discussion

The psychedelic compound 2,5-dimethoxy-4-bromophenethylamine, commonly known as 2C-B, acts as a partial agonist at the 5-hydroxytryptamine-2A (5-HT2A), 5-hydroxytryptamine-2B, and 5-hydroxytryptamine-2C receptors. It also has an affinity for alpha-adrenergic receptors. The effects of 2C-B in humans are primarily due to its modulation of serotonergic and noradrenergic receptors, leading to both physiological and psychological changes. The hallucinogenic properties of 2C-B are mainly attributed to its interaction with the 5-HT2A receptor. The drug’s effects are dose dependent, with a half-life of approximately 1.43 hours, and they typically last between six and 12 hours [12,13].

The impact of 2C-B use is influenced not only by the substance itself but also by the psychological state of the individual. There is a well-established link between substance abuse and bipolar disorder, with individuals suffering from bipolar disorder exhibiting the highest prevalence of substance abuse among major psychiatric disorders [14]. Although the reasons for this co-occurrence are not fully understood, four main theories have been proposed: (a) substance abuse as a symptom of bipolar disorder; (b) substance abuse as an attempt to self-medicate; (c) substance abuse as a cause of bipolar disorder; and (d) substance abuse and bipolar disorder sharing common risk factors [15].

Our patient presented to the emergency department with increased agitation and aggression toward her mother. Her mother reported worsening mood symptoms, recent manic behavior, impulsivity, and risk-taking behaviors, leading to a diagnosis of bipolar disorder I. The patient admitted to frequent party attendance, which increased her exposure to substances, and noted noncompliance with her medication. While the exact motivation for the patient’s use of 2C-B is unclear, it is likely related to her bipolar disorder, given her history of noncompliance and frequent substance use at parties. Research on 2C-B use in bipolar disorder patients is limited, as these individuals are often excluded from studies due to the risk of substance-induced mania. Clinicians should be aware of the risks associated with 2C-B, particularly its rising prevalence and its impact on individuals with psychiatric disorders, especially bipolar disorder.

On June 2, 1995, the Drug Enforcement Administration classified 2C-B as a Schedule I controlled substance. It is commonly encountered in club scenes and distributed through MDMA networks. A typical dose of 2C-B is 10 mg in a capsule or tablet, costing between $10 and $30. It can be ingested orally or snorted in powder form, with snorting often resulting in more intense effects. Lower doses, around 4 mg, produce effects similar to MDMA, such as reduced tension and anxiety. In contrast, higher doses, between 8 mg and 10 mg, amplify stimulating effects and can lead to an intoxicated state. Doses of 20-30 mg often induce hallucinations, while higher doses can result in unpleasant hallucinations and morbid delusions similar to those caused by lysergic acid diethylamide. Given that 2C-B is clandestinely produced, users often lack precise knowledge of the dose they are taking, which can lead to hospitalizations and even death [16].

Detecting 2C-B in patients presents challenges for clinicians, as standard urine screens do not specifically identify it. Although its chemical structure resembles amphetamines, it is unclear at what dose 2C-B might trigger a positive result for amphetamines on a urine drug screen [16]. This complicates the clinical management of patients with recent 2C-B use.

## Conclusions

This case report sheds light on the relatively unknown illicit drug known as 2C. In summary, 2C is a compound that combines stimulant and hallucinogenic properties, presenting a potential risk for abuse, especially within party scenes. The challenges in diagnosing 2C use arise from its nonstandard dosing formulations and the limitations of routine urine testing, which may not accurately identify this substance. Therefore, clinicians should maintain a broad differential diagnosis when assessing patients for potential recreational drug use. Specifically, inquiring about synthetic drug use is crucial, as it helps inform management decisions when routine drug screens may not detect such substances. Further research into the pharmacology, toxicology, and long-term effects of 2C compounds is needed to develop evidence-based strategies for prevention and harm reduction.
